# Editorial: Precision medicine and immune monitoring for infectious diseases

**DOI:** 10.3389/fcimb.2024.1376238

**Published:** 2024-02-15

**Authors:** Jia Li, Liangjing Lu

**Affiliations:** Department of Rheumatology, Ren Ji Hospital, Shanghai Jiao Tong University School of Medicine, Shanghai, China

**Keywords:** precision medicine, infection, immune response, biomarker, therapy

With major clinical and economic repercussions, infections pose a worldwide medical issue. Both the innate and adaptive immune systems are activated by infections in our immune systems. Individuals with compromised immune systems, whether from acquired or intrinsic immunodeficiency, are more vulnerable to opportunistic infections. Conversely, an infection may disrupt immune homeostasis, resulting in excessive inflammation and tissue damage. Researchers and physicians are actively investigating pathogen detection and immune response in infections to address this. The development of targeted biological agents and small molecule compounds has provided new avenues for our understanding of immunological mechanisms and the efficient treatment of infections.

In this Research Topic, reports ranged from the identification of infection and, the exploration of biomarkers to treatment studies. Sun et al. investigated the expression profiles of circular RNAs (circRNAs) in bronchoalveolar lavage fluid (BALF) exosomes of Acute Respiratory Distress Syndrome (ARDS) patients with severe pulmonary infections. Severe pulmonary infections can give rise to ARDS, which is associated with a dismal prognosis. Current treatments for ARDS primarily focus on managing the underlying disease that triggers ARDS and providing supportive care to improve gas exchange and prevent complications. The authors identified two circRNAs, circ042882 and circ104034, which exhibited significant expression differences in BALF exosomes compared to the control group. Targeted mRNA analysis revealed that these circRNAs were mainly enriched in response to hypoxia and decreased oxygen signalling levels. This study highlights the potential role of circRNAs in the pathogenesis of ARDS. There is a dearth of effective therapies that can regulate the excessive inflammatory response in the acute phase of ARDS. Further research is warranted to explore the expression and function of BALF circRNAs in ARDS patients with diverse underlying diseases, which could potentially unravel molecular mechanisms involved in ARDS and lead to the development of new diagnostic or therapeutic strategies.

Neroimmune crosstalk in the inflammatory process has garnered substantial attention. Boshen Yang et al. conducted a study to explore the relationship between the use of 5-hydroxytryptamine-3 receptor (5-HT3R) antagonists, specifically ondansetron (OND), and clinical outcomes in patients with sepsis. The investigation utilized data from the Medical Information Mart for Intensive Care IV (MIMIC-IV) database. The results demonstrated that patients who received OND medication exhibited lower rates of in-hospital, 28-day, and 90-day mortality. Remarkably, the protective effect of OND was observed across various patient subgroups, including different age groups, septic shock cases, vasopressin use, and mechanical ventilation. These findings imply that OND might hold potential as a beneficial intervention in the treatment of sepsis. Notably, 5-HT3R antagonists have been clinically approved due to their anti-inflammatory properties, which include modulating the production of inflammatory cytokines like IL-1β and IL-6 (Boshen et al., 2023; Hur et al., 2014; Maehara et al., 2015; Tsukamoto et al., 2017; Vega Lde et al., 2005) and inhibiting the activation of T cells and macrophages (Boshen et al., 2023; Hur et al., 2014; Maehara et al., 2015; Tsukamoto et al., 2017; Vega Lde et al., 2005). Severe systemic inflammation and organ dysfunction triggered by microbiological agents are hallmarks of sepsis. A significant obstacle that still exists for sepsis is the absence of specific medications. This study provides novel evidence in favour of the potential application of a 5-HT3R antagonist in the management of sepsis.

There are two case reports in this Research Topic. Deng et al.’s report focuses on a case of intestinal Behcet’s syndrome, characterized by abdominal symptoms and complicated by the formation of an entero-urinary fistula and urinary tract infection. The author highlights the effectiveness of anti-inflammatory treatment including the use of biological agents for handling the condition during its acute stage, in conjunction with surgical interventions. Actually, the utilization of biological agents and small molecule compounds has represented a significant milestone in the treatment of autoimmune rheumatic diseases (ARDs). However, it is important to note that concomitant infections frequently occur as complications in ARDs, posing challenges in the management of autoimmune rheumatic diseases (ARDs) and the prevention of infections.

Another study by Chen et al. illustrates the effective combination of metagenomic next-generation sequencing (mNGS) and endobronchial ultrasound-guided transbronchial needle aspiration (EBUS-TBNA) for diagnosing Talaromyces marneffei infection. mNGS provides a quick and highly sensitive method to identify pathogens in clinical settings. Further testing reveals a high titer of plasma anti-interferon-gamma (IFN-γ) autoantibodies in the patient, which is associated with recurrent and/or persistent infections, such as disseminated nontuberculous mycobacterial (dNTM) and viral infections (Chi et al., 2013) (Lin et al., 2016). The application of antifungal medication results in the alleviation of symptoms. Immunocompromised patients are frequently affected by opportunistic fungal infections. In patients with anti-IFN-γ autoantibody (AIGA) positivity, recurrence and/or persistent infections are commonly observed (Qiu et al., 2022) (Shih et al., 2021), Studies have demonstrated a high prevalence of neutralizing anti-IFN-γ autoantibodies in severe T. marneffei infections, particularly in HIV-negative adults (Guo et al., 2020). The presence of anti-IFN-γ autoantibodies is strongly associated with specific HLA alleles (Guo et al., 2020). The specific therapy for anti-IFN-γ autoantibody was not stated in this case, while immunotherapy has occasionally shown promise with treatments such as B-cell depletion or cyclophosphamide (Qiu et al., 2022) (Browne et al., 2012). Investigations into anti-cytokine autoantibody-associated immunodeficiency advance our knowledge of how the host defends itself against particular pathogens (Browne, 2014).

The deployment of new technologies, such as the Biochip, in the field of microbiology, coupled with our deep understanding of the underlying immune mechanisms, has ushered in a new era in the development of precise treatment strategies. As we persist in exerting significant effort, we are tuned up and ready to embrace this new era.

## Author contributions

JL: Writing – original draft. LL: Writing – review & editing.

## References

[B1] Boshen Y. Yuankang Z. Taixi L. Kaifan N. Zhixiang W. Liang L.. (2023). Effects of ondansetron treatment on outcomes of critically ill patients with myocardial infarction partly through its anti-inflammatory activity. Int. J. Med. Sci. 20, 709–716. doi: 10.7150/ijms.81797 37213673 PMC10198140

[B2] Browne S. K. (2014). Anticytokine autoantibody-associated immunodeficiency. Annu. Rev. Immunol. 32, 635–657. doi: 10.1146/annurev-immunol-032713-120222 24499273

[B3] Browne S. K. Zaman R. Sampaio E. P. Jutivorakool K. Rosen L. B. Ding L.. (2012). Anti-CD20 (rituximab) therapy for anti-IFN-γ autoantibody-associated nontuberculous mycobacterial infection. Blood 119, 3933–3939. doi: 10.1182/blood-2011-12-395707 22403254 PMC3350360

[B4] Chi C. Y. Chu C. C. Liu J. P. Lin C. H. Ho M. W. Lo W. J.. (2013). Anti-IFN-γ autoantibodies in adults with disseminated nontuberculous mycobacterial infections are associated with HLA-DRB1*16:02 and HLA-DQB1*05:02 and the reactivation of latent varicella-zoster virus infection. Blood 121, 1357–1366. doi: 10.1182/blood-2012-08-452482 23243276

[B5] Guo J. Ning X. Q. Ding J. Y. Zheng Y. Q. Shi N. N. Wu F. Y.. (2020). Anti-IFN-γ autoantibodies underlie disseminated Talaromyces marneffei infections. J. Exp. Med. 217(12):e20190502. doi: 10.1084/jem.20190502 32880631 PMC7953730

[B6] Hur W. Lee M. K. Park H. P. Kim C. S. Yoon H. J. Zuo Z.. (2014). Ondansetron attenuates the activity of excitatory amino acid transporter type 3 expressed in Xenopus oocytes. Eur. J. Pharmacol. 733, 7–12. doi: 10.1016/j.ejphar.2014.03.027 24690261

[B7] Lin C. H. Chi C. Y. Shih H. P. Ding J. Y. Lo C. C. Wang S. Y.. (2016). Identification of a major epitope by anti-interferon-γ autoantibodies in patients with mycobacterial disease. Nat. Med. 22, 994–1001. doi: 10.1038/nm.4158 27525523

[B8] Maehara T. Matsumoto K. Horiguchi K. Kondo M. Iino S. Horie S.. (2015). Therapeutic action of 5-HT3 receptor antagonists targeting peritoneal macrophages in post-operative ileus. Br. J. Pharmacol. 172, 1136–1147. doi: 10.1111/bph.13006 25377620 PMC4314201

[B9] Qiu Y. Fang G. Ye F. Zeng W. Tang M. Wei X.. (2022). Pathogen spectrum and immunotherapy in patients with anti-IFN-γ autoantibodies: A multicenter retrospective study and systematic review. Front. Immunol. 13. doi: 10.3389/fimmu.2022.1051673 PMC9772057 36569827

[B10] Shih H. P. Ding J. Y. Yeh C. F. Chi C. Y. Ku C. L. (2021). Anti-interferon-γ autoantibody-associated immunodeficiency. Curr. Opin. Immunol. 72, 206–214. doi: 10.1016/j.coi.2021.05.007 34175547

[B11] Tsukamoto A. Sugimoto T. Onuki Y. Shinoda H. Mihara T. Hori M.. (2017). The 5-HT3 receptor antagonist ondansetron attenuates pancreatic injury in cerulein-induced acute pancreatitis model. Inflammation 40, 1409–1415. doi: 10.1007/s10753-017-0584-7 28493078

[B12] Vega Lde L. Muñoz E. Calzado M. A. Lieb K. Candelario-Jalil E. Gschaidmeir H.. (2005). The 5-HT3 receptor antagonist tropisetron inhibits T cell activation by targeting the calcineurin pathway. Biochem. Pharmacol. 70, 369–380. doi: 10.1016/j.bcp.2005.04.031 15922994

